# The HEARTS ECG workshop: a novel approach to resident and student ECG education

**DOI:** 10.1186/s12245-023-00559-0

**Published:** 2023-11-06

**Authors:** Mazen El-Baba, Jesse McLaren, Niran Argintaru

**Affiliations:** 1 Division of Emergency Medicine, Department of Medicine, University of Toronto, Toronto, ON Canada; 2 Division of Emergency Medicine, Department of Family and Community Medicine, University Health Network, Toronto, ON Canada; 3 Department of Emergency Medicine, University of British Columbia, Victoria, BC Canada

**Keywords:** Innovations in EM education, ECG, Medical education

## Abstract

**Objectives:**

ECG interpretation is a life-saving skill in emergency medicine (EM), and a core competency in undergraduate medical curricula; however, confidence for residents/students is low. We developed a novel educational intervention—the HEARTS ECG workshop—that provides a systematic approach to ECG interpretation, teaches EM residents through the process of teaching medical students and highlights emergency management.

**Methods:**

We used the Kern Approach to Curriculum Development. A review of ECG education literature and a targeted needs assessment of local students/residents led to goals and objectives including systematic ECG interpretation with clinical relevance. ECGs were selected based on a national consensus of EM program directors and categorized into 5 common emergency presentations. The educational strategy included content based on HEARTS approach (Heart rate/rhythm, Electrical conduction, Axis, R-wave progression, Tall/small voltages, and ST/T changes), and methods including flipped classroom and near-peer teaching. Evaluation and feedback were based on the Kirkpatrick program evaluation. The workshop was piloted with 6 junior EM residents and 58 medical students, and repeated with nine residents and 68 students from four medical schools.

**Results:**

Residents and students agreed or strongly agreed that the workshop improved their perceived ability (100% and 95%, respectively) and confidence (77% and 88%, respectively) in interpreting ECGs. Reports of ECG interpretation causing anxiety declined from pre-workshop (61% and 83% respectively) to post-workshop (38% and 37% respectively). Residents reported behavior change: 3 months after the workshop, 92.3% reported ongoing use of the HEARTS approach clinically and through teaching medical students on shifts. Reported workshop strengths included the pre-workshop material, the clinical application, facilitator-to-learner ratio, interactivity, the ease of remembering and applying the HEARTS mnemonic, and the iterative application of the approach. Suggested changes included longitudinal sessions with graded difficulty, and allocating more time for introductory material for ease of understanding.

**Conclusion:**

The HEARTS ECG workshop is an innovative pedagogical method that can be adapted for all levels of training. Future directions include integration in undergraduate medical and EM residency curricula, and workshops for physicians to update ECG interpretation skills.

## Introduction

ECG interpretation is a core clinical competency that should be acquired during undergraduate medical training and nurtured during residency [1]. It is critical in emergency medicine (EM) to guide the time-sensitive management of critically ill patients [2]. Development of competency in ECG interpretation relies on mastering an understanding of ECG lead placements, pathophysiological correlation with ECG findings, and clinical relevance of combined ECG information related to rate, rhythm, axis, electrical conduction, electrical amplitude, and ST/T changes [3–5].

Although ECG interpretation is recognized as an essential clinical skill, there continues to be a lack of standardization of ECG teaching [3]. As a consequence, most clinicians do not feel confident in interpreting ECGs, and most medical learners have low accuracy in ECG interpretation [3–7]. Traditional forms of ECG teaching lack innovation and rely on pattern recognition without understanding underlying pathophysiology or clinical relevance [3]. Achieving competency at the post-graduate level is largely reliant on the resident’s self-studying and clinical exposure to ECGs. With multiple competing learning interests and a busy clinical schedule, residents may be disadvantaged in acquiring the foundational knowledge and skills needed to achieve ECG competency, resulting in anxiety related to ECG interpretation [1]. The multiple complexities of ECG interpretations have led many learners to rely on automated computerized interpretation or artificial intelligence (AI)-based interpretation [8]. However, automated interpretation still requires clinician proficiency to interpret and act on information provided for critical diagnoses [9–11].

For medical learners to develop competency in ECG interpretation, a shift in the pedagogical approach is required that incorporates the aforementioned ECG fundamentals and goes beyond basic pattern recognition [12, 13]. To address these challenges and create a competency-based approach to ECG interpretation, we developed the HEARTS ECG workshop—a locally developed, systemic approach to ECG interpretation reinforced through the mnemonic HEARTS (Heart rate/rhythm, Electrical conduction, Axis, R-wave progression, Tall/small voltages, and ST/T changes). The goal of the workshop was to teach a systematic approach to ECG interpretation, enable resident learning through teaching, and link ECG interpretation to the clinical management of emergency department patients.

## Methods

We used the Kern Six-Step Approach to Curriculum Development [14]. (1) *Problem identification* began with a review of ECG education literature and the current local ECG curriculum, which revealed a lack of standardization in teaching and unclear objectives based on learners’ level of training. Although these problems were seen across all levels of training (undergraduate, post-graduate, and in-practice training), the goals of the program targeted undergraduate and junior Emergency Medicine post-graduate medical learners. The program’s goals and objectives and a systematic approach to ECG interpretation were developed based on feedback obtained from the second step of the Kern-Approach—Targeted Needs Assessment.

(2) *Targeted needs assessment* then surveyed medical students and EM residents at our institution, the University of Toronto, to understand their current gaps in their ECG learning and preference in the mode of delivery. This took place in two phases; the first took place 3 months prior to the workshop, which helped prepare the general material. The local medical school class was polled to gauge their interest in an ECG workshop and suggestions for material. The second was completed 1 week before the workshop to poll the participants for their feedback. Themes that emerged from these surveys included the need for more dedicated teaching using a systematic approach to ECG interpretation, developing an approach to identify common and emergent diagnoses, and learning how ECGs can be used in the workup of common emergency presentations including syncope, ischemia, and arrhythmias.

Then, all participants were sent and responded to pre-workshop surveys (13 residents and 126 medical students). All residents (100%) and a majority of medical students (88%) believe that interpreting ECGs is an essential skill for physicians. But a majority of residents and medical students indicated they had received less than 15 h of dedicated ECG training in their undergraduate medical training (70% and 87% respectively); a majority felt this was inadequate (85% and 79%, respectively); and a majority did not feel confident interpreting ECGs (69% and 80%, respectively). Further, survey respondents also indicated that interpreting ECGs in a clinical setting makes them feel anxious (61% and 83%, respectively). Qualitative survey responses requested a step-by-step approach to ECGs and pearls to guide clinical decision-making. Common themes that emerged from both residents and medical students is the need for iterative application of an ECG approach to consolidate their knowledge and build an understanding of ECG interpretation based on pathophysiology and clinical relevance. There was also an emphasis on interactive, small-group learning over didactic lectures or self-directed modules. Residents indicated that they hoped to gain competency in ECG interpretation through teaching and gain confidence in teaching complex concepts to others using a simplified systematic approach.

These informed the program’s (3) *goals and objectives*. Broad goals included learning a systematic approach to ECG interpretation, and understanding how this can help guide emergency management. Specific objectives included learning the HEARTS approach to ECG interpretation, applying this to 5 common emergency presentations, and specific objectives for each. First, 20 ECGs were selected from a consensus of Canadian EM residency program directors for what EM residents need to know [2]. To reinforce clinical relevance, ECGs were then categorized into five frequently encountered clinical presentations in the ED (i.e., palpitations, weakness, syncope, shortness of breath, and chest pain). Then ECGs were interpreted systematically through the HEARTS approach and linked with clinical management—including pitfalls and pearls for each category of cases. Table 1 provides an overview of the workshop materials, including the specific objectives for each category of presentations.

**Table 1:** HEARTS ECG workshop overview

Workshop blocks	Clinical presentations	Learning points
Introduction: HEARTS approach to ECGs (35 min)	Didactic teaching	• ECG basics • Cardiac anatomy • ECG correlates with pathophysiology • HEARTS approach
Palpitations (40 min)	• Supraventricular tachycardia • Ventricular tachycardia • Atrial fibrillation with rapid ventricular response • Wolf-Parkinson-White syndrome	• Develop a differential for tachycardia (wide, narrow, regular, irregular) • ECG application in the approach to stable vs. unstable patients • Recognizing WPW and its clinical significance
Weakness (40 min)	• Hyperkalemia • Infero-posterior occlusion Myocardial Infarction • Atrial Flutter with slow ventricular response • Complete heart block	• Identifying ECG findings of Hyperkalemia and appropriate clinical management • Recognizing emergent causes of bradycardia • Recognizing ECG correlates related to demand ischemia • Identifying appropriate clinical management of causes of bradycardia
Break 30 min		
Syncope (40 min)	• Bifascicular block • Hypertrophic Cardiomyopathy • Brugada syndrome • Prolonged QT	• Develop a systematic approach to syncope based on ECG correlates • Understand the cardiac conduction system and pathophysiology associated with ECG findings (AV block, fascicular block) • Recognizing congenital vs structural causes of syncope and their ECG correlates • Approach to prolonged QT
Shortness of breath (40 min)	• Acute pulmonary embolism with right heart strain • Pericardial effusion • Posterior occlusion Myocardial infarction • Normal left bundle branch block in patient with COPD	• Develop a differential for shortness of breath • Identify ECG correlates of acute right heart strain associated with pulmonary embolism • Identify ECG correlates of tamponade • Understand how POCUS can complement ECG findings
Chest pain (40 min)	• Pericarditis • Benign early repolarization • Left ventricular hypertrophy with secondary repolarization abnormalities • Anterior occlusion Myocardial Infarction	• Develop a differential diagnosis for ST-segment elevation, and differentiate between secondary and primary causes • Differentiate between benign vs. concerning ST-segment elevation • Identify ECG features of pericarditis

(4) *Educational strategy* included content based on the HEARTS approach, and methods including flipped classrooms and learning through teaching [15, 16]. The HEARTS approach begins with an analysis of the Heart rate and rhythm, including how to identify sinus rhythm and diagnose arrhythmias. Second, Electrical conduction assesses the intervals (PR, QRS, QT), and identifies various abnormalities (e.g., WPW, AV blocks, bundle branch blocks, long QT). Third, the limb leads are used to determine the Axis and differentiate different causes of right, left, or extreme axis deviation. Fourth, the precordial leads are used to determine R-wave progression and differentiate different causes of early progression (e.g., RVH, posterior MI, left-sided WPW) or late progression (e.g., anterior infarct). Fourth, the limb and precordial leads are used to assess Tall/small voltages and to differentiate between different causes of tall (e.g., LVH, BER), or small voltages (e.g., pericardial effusion, COPD). Finally, ST/T analysis is deliberately put at the end of interpretation, both to reinforce that these changes can be secondary to abnormalities in rate/conduction/voltage and to help differentiate between secondary and primary changes. This is followed by a summary that consolidates the findings and then puts it in the context of the patient to guide management.

This content was delivered through a flipped classroom method including pre-workshop didactic material, and small group workshop based on near-peer teaching. Pre-workshop preparation material was provided to medical students and EM residents that introduced them to the HEARTS approach. A 15-page PDF was emailed to all participants to read in advance of the workshop. This covered the basics of ECGs including the foundations of cardiac electrophysiology and cardiac anatomy, pictorial correlations with pathophysiology, ECG lead placements, ECG intervals and segments, and then a description and rationale of the HEARTS approach.

Junior EM residents (first and second year) were recruited to facilitate the workshop, under the supervision of an EM physician. EM residents used a pre-workshop manual and teaching slides to learn the approach and to prepare to facilitate the workshop. The teaching slides included a summary of the learning point for each common presentation, and then each of the 20 ECGs was broken down into an individual slide for each component of Heart rate, Electrical conduction, Axis, R-wave progression, Tall/small voltages, and ST/T changes. This was followed by a slide integrating the findings and linking them to emergency management. Residents engaged in a one-hour preparatory session, 1 week prior to the workshop for an overview of the materials and answer questions. It was emphasized that while there would be an EM physician available to oversee the teaching and answer any questions, the residents themselves would be responsible for learning the material to the point of being able to teach it to students—so that residents would learn through the process of preparing and delivering educational material.

The workshop began with an introductory first session provided by the EM physician, which further didactically reinforced the pre-workshop material. This provided all learners the opportunity to ask questions and understand the approach prior to its iterative application to clinical cases.

**Fig. 1:**
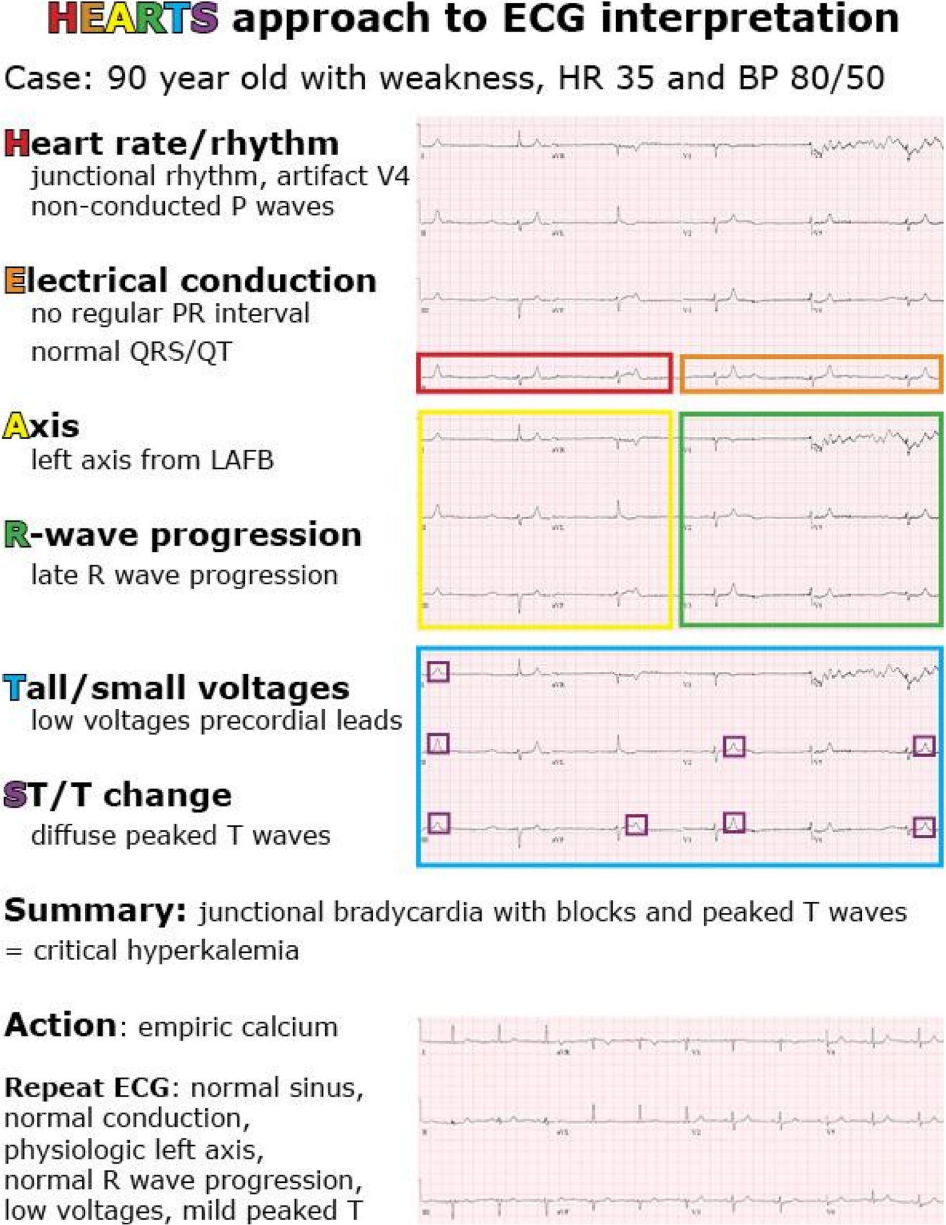
Example of the HEARTS approach and its application on an ECG

The remainder of the workshop constituted small learner groups, each facilitated by a resident. Within the groups, students were asked to interpret the ECG in a step-by-step fashion based on the HEARTS approach. Students would take turns going through each component of the ECG: one student would interpret Heart rate/rhythm, then the next would interpret Electrical conduction, etc. Residents then used slides to highlight both normal and abnormal findings, linked to underlying pathophysiology and clinical relevance. Findings were then amalgamated onto a final slide that allowed students to highlight significant findings and a summary interpretation, which was then linked to clinical management (example shown in Fig. 1) and a discussion of pitfalls and pearls. This process allowed for near-peer teaching and benefited both the facilitator and the learner by learning, applying, and consolidating a systematic approach to ECG interpretation using a repetitive cycle to reinforce and consolidate the HEARTS approach. In other words, the vast majority of the workshop involved residents teaching medical students, learning through the process of teaching. The role of the supervising EM physician was to prepare the pre-workshop material, review all the workshop slides with residents in advance to ensure they were able to teach the material and circulate between the small groups during the workshop to address any questions from medical students for which the residents required further clarification.

(5) *Implementation* included 1 hour pre-workshop reading and a 5-h virtual workshop with supervised small group teaching with an average of 5:1 student-to-facilitator ratio. The workshop was piloted with 6 junior EM residents and 58 medical students. After evaluation and feedback, it was repeated and expanded to nine junior EM residents and 68 medical students from four medical schools, resulting in a total of 15 residents and 126 medical students (16 s-year, 91 third-year, and 19 fourth-year students).

## Results

(6) *Evaluation and feedback* included resident/student surveys, using Likert scales and qualitative questions for Kirkpatrick program evaluation [17]. All residents (*N* = 13) and a third of participating medical students (*N* = 42) responded to the post-workshop survey. Results are presented in Table 2.

**Table 2:** Post-workshop survey results

Question	Medical students	EM residents
The workshop length was appropriate	95% agree or strongly agree	68% agree or strongly agree
The workshop content was appropriate	77% agree or strongly agree	100% agree or strongly agree
The content was clinically relevant	100% agree or strongly agree	100% agree or strongly agree
The workshop improved my ability to interpret ECGs in clinical settings	100% agree or strongly agree	95% agree or strongly agree
Interpreting ECGs makes me feel anxious	37% agree or strongly agree	38% agree or strongly agree

Overall*,* participants had a positive *reaction*: residents and medical students agreed or strongly agreed that the workshop improved their perceived ability and confidence in interpreting ECGs and that the material was clinically relevant. While more medical students found the workshop length was appropriate, more residents found that the content was appropriate to their level. After the workshop, 38% of residents and 37% of medical students endorsed that interpreting ECGs makes them feel anxious (compared with 61% and 83%, respectively, before the workshop). Residents reported *behavior* change when polled 3 months after the workshop, with 92.3% reporting ongoing use of the HEARTS approach clinically and through the teaching of medical students on clinical shifts.

Qualitative feedback from medical students reported that the pre-workshop material, the clinical application of ECG findings, facilitator-to-learner ratio, interactivity, the ease of remembering and applying the HEARTS mnemonic, and the iterative application of the approach were important strengths of the HEARTS ECG workshop. Similarly, residents reported that the group sizes, diversity of clinical presentation, and ease of following the teaching material were important strengths. Residents reported that reviewing the material in advance and having an EM physician available to oversee and answer questions provided both the motivation and the support to learn through the process of teaching.

Suggested changes by medical students to improve the workshop included the creation of longitudinal sessions with graded difficulty and allocating more time for introductory material for ease of understanding. Suggested feedback from residents focused on having more dedicated pre-learning time to prepare for the workshop.

## Discussion

ECG educational literature reveals a lack of confidence and competence across all levels of training, which was confirmed by our needs assessment. While ECG interpretation was highly rated as a necessary skill, learners felt they had not received adequate time or a systematic approach. Pre-workshop survey results are consistent with current literature showing that residents and medical learners perceive ECG interpretation as anxiety-provoking and do not feel confident in applying ECGs in clinical settings. Based on local feedback, a clinically relevant workshop was developed that reinforces a step-by-step approach to ECG interpretation. The development of the HEARTS workshop is in line with the suggestion of national EM program directors calling for additional educational strategies to complement practice-based ECG education [2].

Developing a pedagogical approach to ECG interpretation that balances the broad complexities of ECG analysis and the specifics to improve competency is challenging [3, 8]. The HEARTS workshop overcomes this challenge by standardizing the approach to ECG interpretation and relating the approach to clinical presentations. The measure of this success was evident in the qualitative feedback that was gathered from post-workshop surveys: both residents and medical students reported greater confidence in interpreting ECGs and applying them in clinical settings with less anxiety.

The HEARTS workshop was also useful for EM residents in both its content and delivery. Teaching is an active form of learning that allows residents to self-reflect on knowledge and skills they are lacking with the opportunity to self-improve [18]. Teaching as a form of learning positively impacts residents’ perception of their clinical competency [19] by allowing them to master the learning outcomes to be able to explain them to other medical learners. This process is also advantageous to medical students as near-peer learning has added benefits that improve the learning of clinical skills [20]. Similarly, the supervised near-peer design of the HEARTS workshop encouraged residents to learn through the process of teaching. Evaluations found not only positive reactions to the workshop but also ongoing behavior change.

## Limitations

The workshop was self-organized, which limited the audience to medical students and EM residents who chose to attend on a weekend outside of their regular lectures. Consequently, the assessment of this educational initiative is not immune to selection bias. Participants’ self-selection due to internal motivation to learn ECG interpretation could have impacted their reception and perceived impact of the workshop, including the self-selection involved in the survey response rate; however, the enthusiastic response to an informal workshop, and the pre- and post-workshop surveys illustrate both the inadequacies of the current curriculum, the desire to learn more, and the impact of the workshop.

Another limitation of the HEARTS ECG educational initiative is that it concentrated all the material into one session, with the same material for both residents and students. As a consequence, fewer medical students than EM residents found the workshop material appropriate to their level and suggested graded difficulty with repetitive exposure.

While there was a positive reaction and behavior change, and self-perceived ability, we did not assess ECG competence, which requires years of experience and ongoing feedback. The next step to overcome these limitations is to integrate the workshop into the formal curricula of both medical students and EM residents, with specific adaptations and assessments for each level.

## Conclusion

Establishing a standardized approach to ECG interpretation is an important first step in improving ECG interpretation competency and its clinical application. The HEARTS mnemonic offers an approach to interpretation that is both systematic and simple to learn, captures essential information from an ECG, and helps translate interpretation into clinical management. The HEARTS ECG workshop offers a novel pedagogical model that improves confidence in ECG interpretation to both medical student learners and EM resident facilitators. Future directions include integration within medical school and EM residency curricula, as well as workshops for EM physicians to update their ECG interpretation knowledge and skills.

## Data Availability

The datasets generated during and/or analyzed during the current study are available from the corresponding author on reasonable request.
